# Implementing a novel programme for nurses and allied health professionals to develop capacity for evidence-informed clinical practice

**DOI:** 10.1177/17449871211013074

**Published:** 2021-08-05

**Authors:** Silvie Cooper, Julie Sanders, Nora Pashayan

**Affiliations:** Lecturer (Teaching), Department of Applied Health Research, University College London, UK; Director of Clinical Research, St Bartholomew’s Hospital, Barts Health NHS Trust, UK; Clinical Professor in Cardiovascular Nursing, William Harvey Research Institute, Queen Mary University of London, UK; Professor in Applied Cancer Research, Department of Applied Health Research, University College London, UK

**Keywords:** clinical research, evidence-based practice, nursing education, research awareness, workforce and employment

## Abstract

**Background:**

Nurses and allied health professionals (AHPs) require skills and support to access, appraise, interpret and use research evidence in clinical practice. We describe the process of designing and implementing the Evidence in Practice (EiP) programme at a UK hospital.

**Methods:**

Key stakeholders were engaged to identify learning needs and priorities in appraising and implementing research evidence. To address these, we designed a multi-strategy bespoke programme of activities.

**Results:**

The programme comprised the development of (a) a visual summary of a research paper, (b) five skills development masterclasses and (c) a six-month mentoring scheme to develop and implement plans for translating evidence into practice.

**Discussion:**

The programme overcame many of the traditional barriers (lack of access, skills and time) to increase engagement of nurses and AHP staff in accessing, reviewing and using evidence in clinical practice.

**Conclusion:**

With clinical leadership support, it is feasible to use a multi-strategy approach to promote and enable nurses and AHPs to use evidence in clinical practice.

## Introduction

Research is essential for the provision of safe and high-quality care (International Council of Nurses, 2012) with research active National Health Service (NHS) Trusts in the UK having improved patient outcomes (Ozdemir et al., 2015). Furthermore, in research active organisations, patients report they have more confidence in staff and are better informed about their condition (Jonker et al., 2020). Nurses face individual (lack of knowledge, awareness of available research evidence and critical appraisal skills) and organisational barriers (time to participate in education and professional development opportunities) in engaging with research evidence use in clinical practice (Black et al., 2015). Nurses who value research are more likely to use research findings in practice (Yoder et al., 2014) and thus research literacy and interpretive skills are needed for engaging in evidence-informed practice (Healey, 2005; Jones et al., 2011; Reviriego et al., 2014). Therefore, strategies to optimise research capability and capacity among nurses have the potential to have considerable impact on healthcare delivery and patient outcomes (Gifford et al., 2014).

Initiatives for enabling health and care staff to develop research skills are needed alongside support for research translation (Corbett et al., 2018; Gee and Cooke, 2018; McCance et al., 2006). Despite research being important, many clinical nurses often report a lack of confidence (Woodward et al., 2007) with clinical knowledge being prioritised over research process knowledge and skills (Gullick and West, 2016) and the vast majority having little or no expectation of being involved in research (van Oostveen et al., 2017). Similar issues apply to allied health professionals (AHPs) and strategy approaches to creating AHP research capacity should be inclusive of individuals, organisations and the broader research environment (Pickstone et al., 2008). As the third largest health and care workforce in the UK, AHPs have a critical contribution to make towards the development and delivery of high-quality, patient-centred clinical research (National Institute for Health Research, 2019). Skills in accessing and appraising research are needed to use evidence in practice (Healey, 2005; Jones et al., 2011; Reviriego et al., 2014). This paper describes the process of designing and implementing the Evidence in Practice (EiP) programme, aimed at supporting nursing and AHP staff to access, appraise, interpret and use research evidence in practice.

## Methods

**Figure 1.**
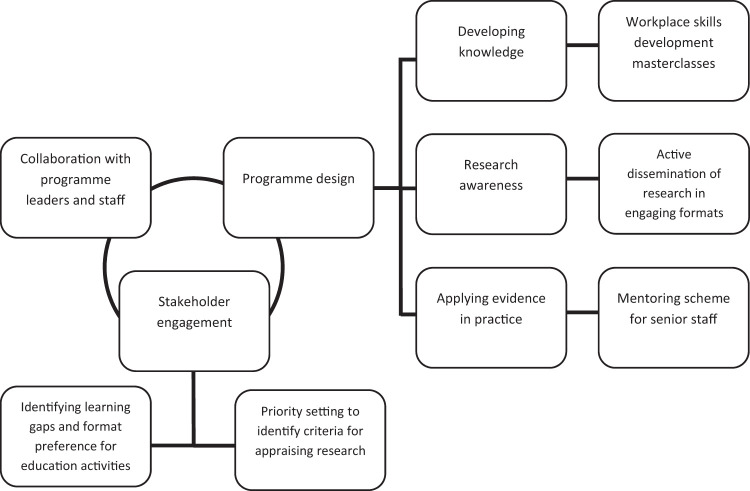
Structure of the Evidence in Practice (EiP) programme.

The EiP programme was a funded joint initiative between a research-intensive university and a specialist hospital in the UK. The hospital has a Director of Clinical Research for nurses, AHP and healthcare science (HCS) staff and provides a range of bespoke research in practice initiatives alongside a clinical academic career programme. A team of researchers, educators and clinicians designed the EiP programme. We drew on literature and stakeholder feedback about engaging healthcare staff to use research evidence in clinical practice. Figure 1 depicts the process of developing the programme.

### Stakeholder engagement: building collaboration, identifying learning needs and priorities for reviewing research evidence

Funding was secured for the initiative from University College London (UCL) Partners Academic Health Science Network (ASHN) and a partner NHS hospital was identified. Stakeholders included those at the directorate level (programme leaders from the hospital and university sites) and staff who participated in the programme. Several meetings with programme leaders from the university and partner NHS Trust were held to explore similar initiatives that had been previously run at the hospital, such as masterclasses and secondment schemes. Through the planning meetings we aimed to establish what had been successful with these initiatives and why, and whether staff had experienced barriers in participating. We sought to equip nurses and AHP staff of all levels to use research evidence in clinical practice, and wanted to ensure the aims of the EiP programme were fit for purpose for the group of staff we were engaging.

Following these planning discussions with programme leaders, we then held a stakeholder event for nurses, AHP and HCS staff, in which we conducted an exploratory discussion about the proposed activities and content of the EiP programme. We wanted to understand staff perspectives of the role of research in their day-to-day clinical practice. In the discussion, we first presented an overview of different formats for reading research evidence. We asked how staff access research evidence, with further prompts for the group to consider where they access this information from, the source, and how frequently this occurs. The group chose context, relevance to practice, methods of the paper, applicability to practice and date of publication as criteria for reviewing research. Working in smaller groups, stakeholders were asked to give each criterion a score from 0 to 100 such that the total score per group would be 100. These scores were then weighted for each criterion by taking the average scores across the groups and dividing by 100 (for instance, ‘context’ had a weighted score of 0.16 and ‘relevance to practice’ was weighted at 0.26). We then asked that they review the title and abstract of several example research papers and give a score between 0 and 1, assessing the applicability of their selected criteria (context, relevance to practice, methods of the paper, applicability to practice, date of publication) to each of the papers. Using these scores and further consultation with stakeholders, we selected one paper to be turned into a visual abstract that was created by a professional illustrator. The visual abstract was part of the ‘research awareness’ component of the programme, which aimed to promote active dissemination of research evidence in clinical practice.

### Designing a multi-strategy, bespoke programme of activities

Following the planning discussions and engagement with stakeholders, we designed a complementary programme of activities, which included: (a) skills development masterclasses; (b) active dissemination of research evidence through the creation of a visual summary of a research paper; and (c) a mentoring scheme.

#### Knowledge engagement: skills masterclasses offered in the workplace

Engaging NHS staff with research can improve their ability, willingness and likelihood of using research evidence (Marjanovic et al., 2019). A lack of confidence to engage with research and skills for using evidence in practice can limit clinical nurses’ participation in research (Gullick and West, 2016; van Oostveen et al., 2017; Woodward et al., 2007). AHPs face similar barriers, and strategies for building research capacity among nursing and AHP staff groups should account for individual, organisational and environmental factors that shape participation and inclusion in research and evidence implementation (Pickstone et al., 2008). Practical skills for accessing and appraising research evidence are needed to participate effectively in interpreting and implementing evidence into practice (Healey, 2005; Jones et al., 2011; Reviriego et al., 2014). From the planning and stakeholder discussions, we identified that practical, interactive sessions focusing on applied learnings related to literature searching, critical appraisal and implementation of research evidence were suitable for this audience of nurses and AHP staff. The site had previously hosted a masterclass series that was well received by staff. We similarly designed a series of small group tutorials offered in the workplace to match this masterclass format, maximising opportunities for staff to attend. We tailored the content further by accounting for the likely audience, the subject of research that would be relevant to them (as staff at a cardiac and cancer specialist hospital), and the brief time and opportunity for learning offered through the masterclasses. The masterclasses were structured around short lectures, small-group exercises, and individual tasks.

#### Research awareness: research evidence disseminated in accessible formats

Having identified a suitable research paper through the priority setting exercise conducted during the stakeholder engagements, we commissioned a professional illustrator to translate the paper into an engaging visual summary. A narrative for the visual summary was developed collaboratively with the illustrator to ensure that the key messages were teachable and accessible to a diverse audience. The aim with this activity was to create a ‘bite-size’, accessible summary, which staff could readily read and understand (Yhnell et al., 2019).

#### Applying evidence: a mentoring secondment scheme for senior AHPs

Through stakeholder engagement, we identified that staff were not able to access practical experience of how to implement research evidence into practice. In response, we designed a mentoring scheme for senior staff to gain experiential learning through working with a mentor on developing an implementation plan for using research evidence in clinical practice (Black et al., 2015; O’Byrne and Smith, 2011; Smith et al., 2014).

## Results and outputs

### Masterclasses

We delivered five hour-long highly interactive small-group masterclasses (topics shown in Table 1), offered on a monthly basis in the workplace by experienced researchers and educators with expertise in implementation, literature searching and evidence appraisal and interpretation. The masterclasses provided staff with information about the approaches and skills they would need to access, appraise and use evidence in practice. Between five and 20 participants attended each masterclass. A feedback form was collected from participants at the end of each masterclass. Feedback was favourable, with most reporting that the learning objectives were clear and appropriate for their level of their understanding.

**Table 1.** Skills development masterclasses topics and learning outcomes.

Topic	Learning outcomes – Participants would know how to:
1. Accessing evidence	Create an answerable research question and access existing research in an area of interest, develop a search strategy and access resources from NHS Trust Library
2. Appraising evidence: tools for appraisal	Assess the validity of a study, critique results and determine the local relevance of findings, and what critical appraisal involves
3. Appraising evidence: applying skills	Apply a framework to critically appraise published research, to find study characteristics in a research paper, and explain if the results are valid
4. Interpreting evidence	Critically read research findings reported in the media and policy and interpret research findings by contextualising them
5. Implementing evidence in practice	Critically examine the evidence that underpins routine clinical practice, explain the practicalities of changing practice and identify local and organisational factors that may support or limit evidence implementation

### Disseminating research evidence

The visual summary was a cartoon illustration that presented the abstract of a paper by Jones et al. (2017) about how hospital boards govern for quality improvement. The paper was identified by staff, who participated in the stakeholder engagement event, as being relevant and of interest to the group for explaining a frequently drawn-on concept (quality improvement) and the practical processes of how governing bodies operate. The visual summary was easily shared as a poster at the site and conferences, and as an email link cascaded to staff at the site.

### Mentoring scheme

Staff at the site were invited to submit an expression-of-interest form detailing their interest and suitability for the scheme. Applications were reviewed by programme leaders to assess that selected participants were senior staff, had an idea for service change or innovation, and would benefit from the opportunity of taking part in the scheme. Two participants were selected, one Perfusionist and an Echocardiographic Research Lead (both senior AHP and HCS staff at the site). Mentors, who had expertise in research, evidence appraisal and innovation and implementation practice, supported them. Each champion met with mentors over the course of the programme to plan a project for implementing an evidence-based change into practice. The champions were enabled to develop leadership skills in learning to communicate their ideas for innovation and practice change to various audiences (colleagues, mentors, managers and multidisciplinary teams), research skills (interpretation and evaluation) and implementation science skills. The champions identified a need for a change in practice and assessed existing evidence to support implementation of the change. They conceptualised what the change might entail (gathering further evidence or developing a standardised way of working, for instance) and built an action plan of next steps of moving towards implementation of the change. One champion aimed to develop a standardised perfusion protocol and guidelines for aortic dissection to reduce variation across clinical practice in the service. The other champion explored the feasibility of adopting a novel application of 3D echo-cardio measurements for non-invasive evaluation of pulmonary circulation and left heart function. In debriefing sessions and a follow up interview with mentoring scheme participants, champions reported working closely with their clinical teams to assess organisational readiness for change. Champions took on leadership roles to build capacity in their teams by organising training opportunities, such as audit day updates on new techniques, and critical appraisal and literature searching sessions offered in collaboration with the hospital’s Clinical Librarian service. One champion reported gaining confidence in influencing for change and being able to guide others towards collaborative working. This champion also strengthened communication between stakeholders and encouraged open dialogue in multidisciplinary teams by bringing an evidence-based focus to decision making alongside clinical experience and expertise. The champions were both able to engage stakeholders collectively, communicate the need for understanding practice and identifying necessary changes to improve outcomes for the service and patients.

## Discussion

We built productive relationships between programme leaders and stakeholders to understand the specific organisational priorities, existing initiatives and learning needs of the group. Taking a multi-strategy approach meant we engaged with diverse groups of staff through the various activities in the programme. We created a bespoke, tailored programme by including stakeholder views and using literature to inform our approach at every stage of developing and implementing this programme. This was to enhance the applicability and relevance of key content and learnings disseminated to staff at the site, and promote use of research evidence in clinical practice (Barratt and Fulop, 2016; Marjanovic et al., 2019).

By delivering the programme in the workplace, and providing mentoring, the EiP programme overcame many of the traditional barriers (lack of access, skills and time) to participating in continuous professional development opportunities (Black et al., 2015; McCance et al., 2006). Presentations and formats that are user-friendly and engaging can facilitate dissemination of research evidence (Black et al., 2015; Yhnell et al., 2019). The masterclass tutorial structure, topics and intended learning outcomes enabled attendees to apply learnings immediately and check their understanding of concepts with peers and with the tutors, and engage in active thinking through think-pair-share strategies (Kaddoura, 2013). Using these strategies in the masterclass exercises allowed us to gauge the group’s level of understanding of the material that had just been taught, and to give feedback and address queries by engaging in a dialogue and collaborative exchange (Cooper and Robinson, 2000). We conceptualised the users of research evidence in this programme to be staff working at various levels of the hospital, from those in frontline care, to those undertaking formal research training and with specific research roles embedded in their clinical roles (Marjanovic et al., 2019). We accounted for organisational and collaboration factors (Marjanovic et al., 2019) in aligning the aims of our programme with the site’s priorities for developing research culture and engaging nurses and AHP staff in research at the site. Gaining buy-in from several levels of the organisation (board, executive, senior and frontline staff) meant that there was support for the programme. This was an important step for drawing on internal organisational infrastructure and processes in designing and implementing programme activities (Gee and Cooke, 2018).

### Lessons to take forward for further iterations of these programmes

The format of a one-hour masterclass delivered in the workplace made it feasible for staff to attend. However, as this was during worktime, clinical duties might have taken priority for staff (Gullick and West, 2016), and attendance was not predictable. Integration of select masterclasses or the series into larger events to raise research awareness and develop research culture at the site (such as open days, audit days and other learning events) might have extended the reach of the programme to staff across the hospital and allowed staff to have protected time to attend and participate.

Visual summaries may be effective as springboards for raising awareness of research evidence, engaging new audiences and driving interest by providing the information in an accessible format (Pedwell et al., 2017; Rodríguez Estrada and Davis, 2015). It is feasible to disseminate relevant research evidence to frontline staff as users of research. Considering how the audience might engage, and what opportunities and forums exist in their workplace to learn about research findings could increase reach of these initiatives. Ambassadors and champions play an important role in reaching staff too, particularly those involved in delivering frontline care, and so early collaboration with these stakeholders might support further dissemination of research findings in clinical practice (Hardicre, 2014; Yhnell et al., 2019).

We faced challenges in engaging staff at the site with the mentoring secondment, both at the point of gaining expressions of interest and in running the secondment. A bespoke programme for ward managers to increase research knowledge, skills and confidence, and to provide positive research environments has previously been conducted in the same trust. Interventions that target those with positional power to support and sustain change in clinical practice have far-reaching effectiveness (Gifford et al., 2014). We had buy-in from senior leaders and the board at the host NHS site, as well as from mentors at the university and in the ASHN who funded the project. However, as the EiP mentoring scheme involved staff taking funded time out from clinical practice, it would have been helpful to work more closely with those in charge of clinical rotas and advance planning in supporting staff to participate and line managers to fill in rotas. Further iterations of these programmes could incorporate early engagement with users, collaborators and mentors and align aims of the secondment with organisational priorities and professional development opportunities to ensure there is support for these schemes from all levels of the organisation, including executive boards, managers, clinical teams, and staff who might participate.

By focusing on research evidence use (rather than production) and active research dissemination of the visual summary in this programme, we address gaps identified by Gee and Cooke (2018) in their review of research capacity development strategies. The programme overcame individual and organisational barriers, such as a lack of research awareness and critical appraisal skills, and time and awareness for participating in education related to research as discussed by Black et al. (2015), by creating diverse opportunities for skills development. Further, senior staff had time out of their clinical role (Black et al., 2015; van Oostveen et al., 2017) to participate in the mentoring scheme to plan for implementing evidence-based service changes into clinical practice. By providing mentoring, the EiP programme utilised an experiential learning model that is reflected in several clinical nursing research capacity building initiatives (O’Byrne and Smith, 2011). The strength of the EiP programme was to take a multi-strategy approach, designed with leaders and stakeholders and aligned with organisational priorities of the host site, which are important requirements for successful capacity-building initiatives (O’Byrne and Smith, 2011) in supporting staff to access, appraise, interpret and use research evidence in practice.

## Conclusion

We built productive relationships between programme leaders and stakeholders to understand the specific learning and organisational needs of the group. Having tailored the design of the programme for point-of-care staff, further consideration could be given to how to extend these programmes and integrate them more clearly with existing initiatives to raise research awareness among nursing and AHP staff and develop research culture in clinical settings. With clinical leadership support, it is feasible to use a multi-strategy approach for educating and enabling nurses and AHPs to use research evidence in clinical practice. By designing a programme that was informed by literature and in consultation with stakeholders, we identified gaps and developed several complementary activities to promote evidence use in healthcare practice through this process. Evaluation of the effectiveness and impact of this approach to designing these kinds of education programmes is needed.

## Key points for policy, practice and/or research

- We built productive relationships between programme leaders and nurses and AHP staff to understand the specific organisational priorities and learning needs of the group.
- By taking a multi-strategy approach, we were able to include stakeholders at every stage of developing and implementing this programme, which enhanced the applicability and relevance of key content and learnings disseminated to staff at the site.
- By delivering the EiP programme in the workplace with clinical leadership support, and providing mentoring, this programme overcame many of the traditional barriers (lack of access, skills and time) to participating in continuous professional development and education opportunities.

## References

[bibr1-17449871211013074] Barratt H Fulop NJ (2016) Building capacity to use and undertake research in health organisations: a survey of training needs and priorities among staff. BMJ Open 6: e012557. 10.1136/bmjopen-2016-012557 PMC5168601 27927657

[bibr2-17449871211013074] Black AT Balneaves LG Garossino C , et al. (2015) Promoting evidence-based practice through a research training program for point-of-care clinicians. Journal of Nursing Administration 45: 14. 10.1097/NNA.0000000000000151 PMC4263611 25390076

[bibr3-17449871211013074] Cooper JL Robinson P (2000) Getting started: informal small-group strategies in large classes. New Directions for Teaching and Learning 2000: 17–24.

[bibr4-17449871211013074] Corbett J D’Angelo C Gangitano L , et al. (2018) Future of health: findings from a survey of stakeholders on the future of health and healthcare in England. Rand Health Quarterly 7: 1. 10.7249/rhq-07-03-01 PMC5873518 29607245

[bibr6-17449871211013074] Gee M Cooke J (2018) How do NHS organisations plan research capacity development? Strategies, strengths, and opportunities for improvement. BMC Health Services Research 18: 198. 29566696 10.1186/s12913-018-2992-2 PMC5865402

[bibr7-17449871211013074] Gifford WA Holyoke P Squires JE , et al. (2014) Managerial leadership for research use in nursing and allied health care professions: a narrative synthesis protocol. Systematic Reviews 3: 1–7. 24903267 10.1186/2046-4053-3-57 PMC4072612

[bibr8-17449871211013074] Gullick JG West SH (2016) Building research capacity and productivity among advanced practice nurses: an evaluation of the Community of Practice model. Journal of Advanced Nursing 72: 605–619. 26537181 10.1111/jan.12850

[bibr9-17449871211013074] Hardicre J (2014) Embedding the 6 Cs into clinical research practice and management. British Journal of Nursing 23: 365–367. 24732988 10.12968/bjon.2014.23.7.365

[bibr10-17449871211013074] Healey M (2005) Linking research and teaching to benefit student learning. Journal of Geography in Higher Education 29: 183–201.

[bibr11-17449871211013074] International Council of Nurses (2012) *Position Statement: Patient Safety.* Available at: https://www.icn.ch/sites/default/files/inline-files/D05_Patient_Safety_0.pdf (accessed 17 August 2019).

[bibr12-17449871211013074] Jones L Pomeroy L Robert G , et al. (2017) How do hospital boards govern for quality improvement? A mixed methods study of 15 organisations in England. BMJ Quality and Safety 26: 978–986. 10.1136/bmjqs-2016-006433 PMC5750431 28689191

[bibr13-17449871211013074] Jones SC Crookes PA Johnson KM (2011) Teaching critical appraisal skills for nursing research. Nurse Education in Practice 11: 327–332. 21474381 10.1016/j.nepr.2011.03.002

[bibr14-17449871211013074] Jonker L Fisher SJ Dagnan D (2020) Patients admitted to more research-active hospitals have more confidence in staff and are better informed about their condition and medication: results from a retrospective cross-sectional study. Journal of Evaluation in Clinical Practice 26: 203–208. 30784152 10.1111/jep.13118

[bibr40-17449871211013074] Kaddoura M (2013) Think pair share: A teaching learning strategy to enhance students' critical thinking. *Educational Research Quarterly* 36(4): 3–4.

[bibr15-17449871211013074] Marjanovic S, Ball S, Harshfield A, et al. (2019) Involving NHS staff in research. Healthcare Improvement Studies Institute, Cambridge. Available at: https://www.thisinstitute.cam.ac.uk/research-articles/involving-nhs-staff-in-research/ (accessed 8 September 2020).

[bibr16-17449871211013074] McCance TV Fitzsimons D Armstrong NC (2006) Developing a best practice framework to benchmark research and development activity in nursing and midwifery. Journal of Research in Nursing 11: 160–171.

[bibr17-17449871211013074] National Institute for Health Research (2019) *NIHR CRN Allied Health Professionals Strategy 2018–2020*. Available at: https://www.nihr.ac.uk/documents/nihr-crn-allied-health-professionals-strategy-2018-2020/11530 (accessed 8 September 2020).

[bibr18-17449871211013074] O’Byrne L Smith S (2011) Models to enhance research capacity and capability in clinical nurses: a narrative review. Journal of Clinical Nursing 20: 1365–1371. 21040029 10.1111/j.1365-2702.2010.03282.x

[bibr19-17449871211013074] Ozdemir BA Karthikesalingam A Sinha S , et al. (2015) Research activity and the association with mortality. PLoS One 10: e0118253. 25719608 10.1371/journal.pone.0118253 PMC4342017

[bibr20-17449871211013074] Pedwell RK Hardy JA Rowland SL (2017) Effective visual design and communication practices for research posters: exemplars based on the theory and practice of multimedia learning and rhetoric. Biochemistry and Molecular Biology Education 45: 249–261. 27925371 10.1002/bmb.21034

[bibr21-17449871211013074] Pickstone C Nancarrow S Cooke J , et al. (2008) Building research capacity in the allied health professions. Evidence and Policy 4: 53–68.

[bibr22-17449871211013074] Reviriego E Cidoncha MÁ Asua J , et al. (2014) Online training course on critical appraisal for nurses: adaptation and assessment. BMC Medical Education 14: 136. 24996951 10.1186/1472-6920-14-136 PMC4107575

[bibr23-17449871211013074] Rodríguez Estrada FC Davis LS (2015) Improving visual communication of science through the incorporation of graphic design theories and practices into science communication. Science Communication 37: 140–148.

[bibr24-17449871211013074] Smith B, Hurth J, Pletcher L, et al. (2014) *A guide to the implementation process: stages, steps and activities*. ECTA Center. Available at: http://ectacenter.org/∼pdfs/implementprocess/implementprocess-stagesandsteps.pdf (accessed 8 September 2020).

[bibr25-17449871211013074] Van Oostveen CJ Goedhart NS Francke AL , et al. (2017) Combining clinical practice and academic work in nursing: a qualitative study about perceived importance, facilitators and barriers regarding clinical academic careers for nurses in university hospitals. Journal of Clinical Nursing 26: 4973–4984. 28793367 10.1111/jocn.13996

[bibr26-17449871211013074] Woodward V Webb C Prowse M (2007) The perceptions and experiences of nurses undertaking research in the clinical setting. Journal of Research in Nursing 12: 227–244.

[bibr27-17449871211013074] Yhnell E Smith HA Walker K , et al. (2019) #WhyWeDoResearch: raising research awareness and opportunities for patients, public and staff through Twitter. Research for All 3: 7–17.

[bibr28-17449871211013074] Yoder LH Kirkley D McFall DC , et al. (2014) CE: Original Research: Staff nurses’ use of research to facilitate evidence-based practice. American Journal of Nursing 114: 26–37. 10.1097/01.NAJ.0000453753.00894.29 25121949

